# Supplementary material for: A Novel Strategy for Identifying NSCLC MicroRNA Biomarkers and Their Mechanism Analysis Based on a Brand-New CeRNA-Hub-FFL Network
Source: Int J Mol Sci. 2022 Sep 25;23(19):11303. doi: 10.3390/ijms231911303 (PMC9569765; doi:10.3390/ijms231911303)
Supplement: Supplementary file 1 [file ijms-23-11303-s001.zip › ijms-1856979-supplementary.pdf]

Supplementary Figures and Tables

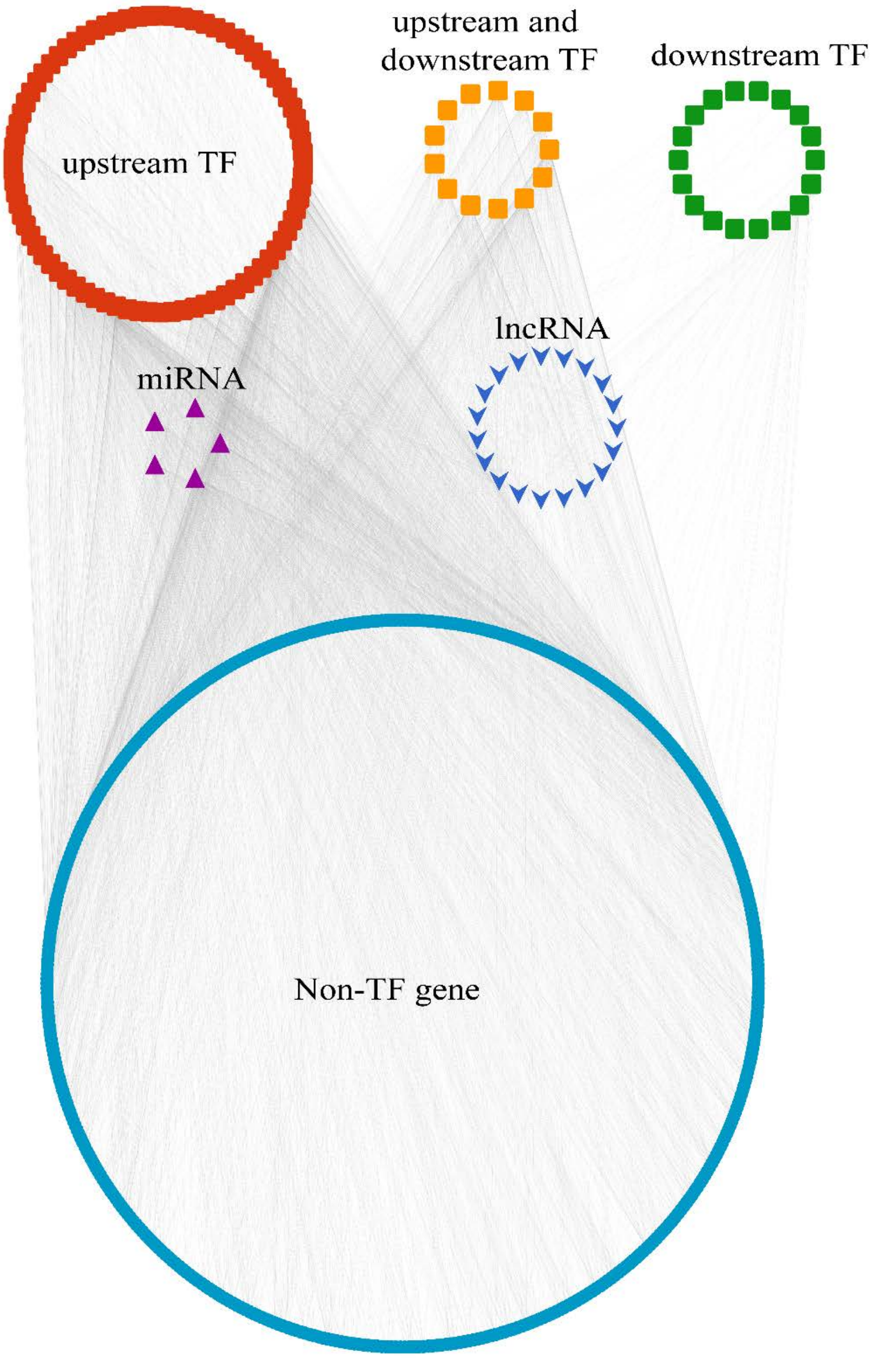

Figure S1. FFL constructed based on miRNA markers.

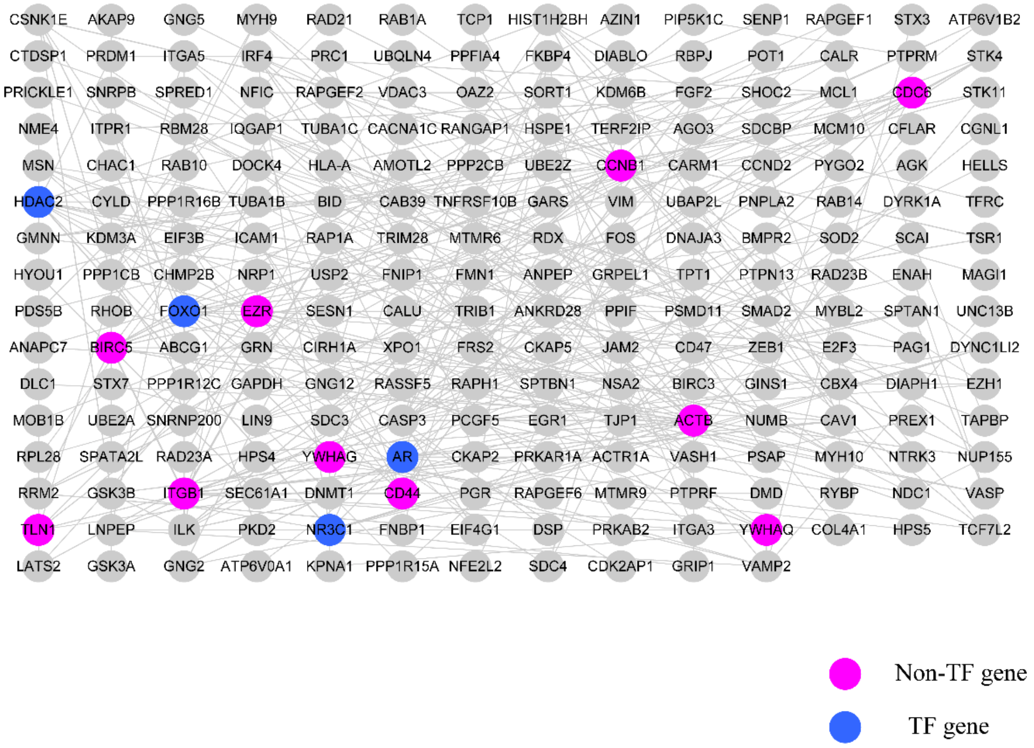

Figure S2. PPI network of miRNA target genes.

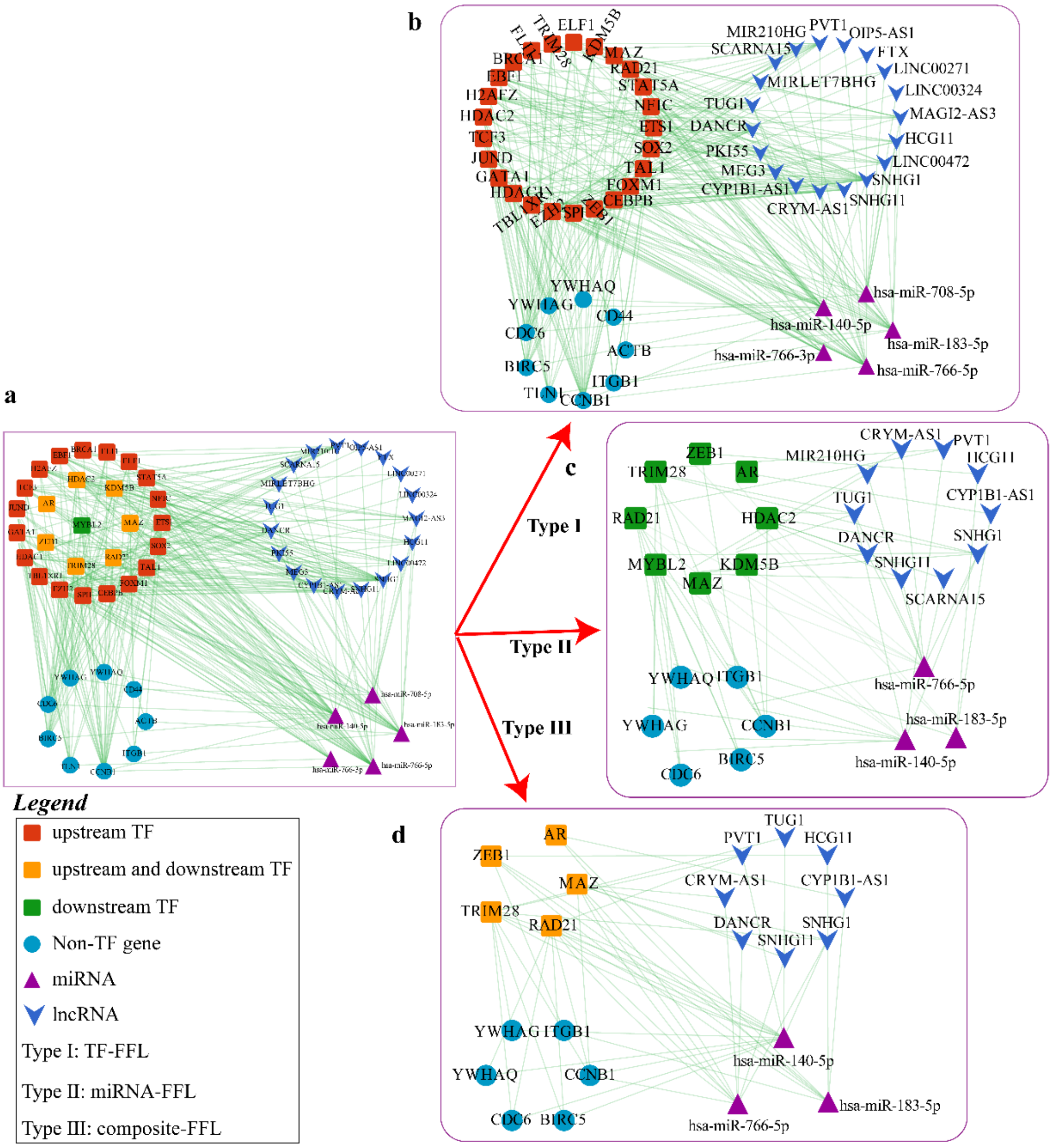

Figure S3. Hub-FFL. Fig. S3a is the total network; it can be divided into three different types of hub-FFL networks. (b)Type I subnetwork. (c) Type II subnetwork. (d) Type III subnetwork.

Table S1. MiRNAs with significantly high independent regulatory capacity in LUAD.

miRNA	NOG value	p-value of NOG	TFP value	p-value of TFP
**miR-218-5p**	12	1.37E-36	0.1638	0.029499
**miR-26a-5p**	11	2.24E-35	0.1656	0.020696
**miR-101-3p**	8	2.24E-27	0.177	2.55E-04
**miR-32-5p**	7	9.22E-24	0.2639	3.55E-15
**miR-296-5p**	7	9.22E-24	0.25	1.78E-14
**miR-96-5p**	6	1.31E-18	0.1692	0.006276
**miR-224-5p**	6	1.31E-18	0.1774	1.97E-04
**miR-708-5p**	6	1.31E-18	0.1628	0.045212
**miR-129-5p**	5	4.29E-13	0.2727	1.78E-15
**miR-150-5p**	5	4.29E-13	0.1667	0.014513
miR-30c-5p	4	2.88E-07	0.1799	5.68E-05
miR-181c-5p	4	2.88E-07	0.194	2.38E-07
miR-2115-5p	4	2.88E-07	0.2222	2.53E-12
miR-618	3	0.023184	0.1905	7.84E-07
miR-1908-5p	3	0.023184	0.2105	7.82E-10
miR-190a-5p	3	0.023184	0.1923	4.36E-07
miR-1260a	3	0.023184	0.25	1.78E-14
miR-192-5p	3	0.023184	0.1875	3.78E-06
miR-216b-5p	3	0.023184	0.1765	3.14E-04
miR-146b-5p	3	0.023184	0.1852	1.04E-05

Note: default threshold: p-value <0.05, Wilcoxon signed-rank test, NOG values of miRNAs bigger than the median are marked as bolded.

Table S2. MiRNAs with significantly high independent regulatory capacity in LUSC.

miRNA ID	NOG value	p-value of NOG	TFP value	p-value of TFP
**miR-766-3p**	16	1.60E-31	0.1587	0.015976

**miR-101-3p**	12	3.30E-27	0.1572	0.019063

Continued Table S2

**miR-296-5p**	10	1.05E-21	0.2281	1.39E-11
**miR-766-5p**	10	1.05E-21	0.1667	0.002393
**miR-744-5p**	8	7.11E-15	0.2133	1.06E-09
**miR-96-5p**	8	7.11E-15	0.1641	0.003513
**miR-1908-5p**	7	5.16E-11	0.2667	1.14E-13
**miR-140-5p**	7	5.16E-11	0.2195	1.25E-10
**miR-183-5p**	7	5.16E-11	0.16	0.010558
miR-532-3p	6	4.34E-07	0.2121	1.37E-09
miR-491-5p	6	4.34E-07	0.2927	5.68E-14
miR-320e	6	4.34E-07	0.2286	9.21E-12
miR-224-5p	6	4.34E-07	0.1782	3.58E-05
miR-219a-5p	6	4.34E-07	0.1667	0.002393
miR-346	5	3.05E-04	0.1842	6.86E-06
miR-19b-1-5p	5	3.05E-04	0.1781	3.78E-05

Note: default threshold: p-value <0.05, Wilcoxon signed-rank test, NOG values of miRNAs bigger than the median are marked as bolded.

Table S3. LUAD-related important genes.

Gene symbol	Gene ID	Gene symbol	Gene ID
ADAMTS8	11095	CBX8	57332
AKIRIN2	55122	CCDC6	8030
ANXA1	301	CDKN1A	1026
AQP1	358	CDON	50937
BAIAP2L1	55971	CEACAM6	4680
CADM1	23705	CRK	1398
CAV1	857	CSF3	1440
CBFA2T3	863	CSNK1A1	1452
CXCL3	2921	MAL	4118
CXXC5	51523	MINA	84864
DCUN1D3	123879	MUC4	4585
DLC1	10395	MXI1	4601
DLX5	1749	NKX3-1	4824

Continued Table S3

DNAJA3	9093	NME1	4830
DNAJB1	3337	NPAS2	4862
DUSP26	78986	NPM1	4869
DUSP9	1852	PAEP	5047
E2F5	1875	PAX6	5080
EDNRB	1910	PDGFB	5155
EEF1A2	1917	PDGFRL	5157
EMP1	2012	PDLIM4	8572
EMP2	2013	PEBP1	5037
ERBB2	2064	PELP1	27043
ETS1	2113	PIK3R1	5295
FADD	8772	PLAGL1	5325
FGF10	2255	PSIP1	11168
FGFR1	2260	PTTG1IP	754
FHL1	2273	RASAL1	8437
FLCN	201163	RASSF1	11186
FOXA1	3169	RASSF8	11228
FOXP1	27086	SFN	2810
FOXQ1	94234	SFRP1	6422
FYN	2534	SGMS1	259230
FZD2	2535	SIK1	150094
GADD45B	4616	SLC39A4	55630
GPR110	266977	SLIT2	9353
GPR132	29933	SMARCA4	6597
GPX3	2878	SOX11	6664
HDAC1	3065	SOX7	83595
HIC1	3090	SPARCL1	8404
HSPA1A	3303	SRC	6714
HTATIP2	10553	TBX5	6910
ID1	3397	THBD	7056
ID2	3398	TP53	7157
IDH1	3417	TRIM35	23087
ING3	54556	TRIT1	54802
JUP	3728	TSLP	85480
KLF4	9314	TWIST2	117581
KLF6	1316	WNK2	65268
LAPTM4B	55353	ZBTB7A	51341

Table S4. LUSC-related important genes.

Gene symbol	Gene ID	Gene symbol	Gene ID
ADAMTS8	11095	DLX5	1749

Continued Table S4

AKAP13	11214	DMBT1	1755
AKAP9	10142	DNAJA3	9093
AQP1	358	DNMT3A	1788
ARL6IP5	10550	DUSP22	56940
BAIAP2L1	55971	DUSP26	78986
BATF2	116071	DUSP9	1852
BCL2L1	598	ECT2	1894
BCL3	602	EDNRB	1910
BHLHE41	79365	EED	8726
BIRC5	332	EEF1A2	1917
CABLES1	91768	EI24	9538
CADM1	23705	EIF3I	8668
CAV1	857	EMP1	2012
CBFA2T3	863	EMP2	2013
CBX8	57332	EPB41L3	23136
CCDC28A	25901	EPHA3	2042
CCNB1	891	ETS1	2113
CCNDBP1	23582	FADD	8772
CDK2	1017	FAM83D	81610
CDKN1A	1026	FBP1	2203
CDON	50937	FGF10	2255
CEACAM6	4680	FGFR1	2260
CMTM3	123920	FHL1	2273
CRK	1398	FLCN	201163
CRLF2	64109	FOS	2353
CSF3	1440	FOXA1	3169
CSNK2A1	1457	FOXA2	3170
CTDSPL	10217	FOXM1	2305
CXCL3	2921	FOXP1	27086
CXCR4	7852	FRAT1	10023
DCN	1634	FUS	2521
DCUN1D1	54165	FYN	2534
DCUN1D3	123879	G0S2	50486
DIABLO	56616	GADD45B	4616
DLC1	10395	GADD45G	10912
GADD45GIP1	90480	MRAS	22808
GFI1	2672	MRE11A	4361
GLTSCR2	29997	MTUS1	57509
GMNN	51053	MUC1	4582
GNAI2	2771	MXI1	4601
GPR110	266977	NCOA3	8202
GPRC5A	9052	NEDD4L	23327
GPX3	2878	NEDD9	4739

Continued Table S4

GSK3A	2931	NINJ1	4814
H2AFX	3014	NKX2-1	7080
HDAC1	3065	NME1	4830
HIC1	3090	NRBP1	29959
HLTF	6596	NUP98	4928
HOMER2	9455	OLR1	4973
HRAS	3265	PA2G4	5036
HSP90B1	7184	PAEP	5047
HSPA1A	3303	PARP1	142
HSPD1	3329	PAX6	5080
HTATIP2	10553	PDCD5	9141
ID1	3397	PDGFB	5155
ID2	3398	PDLIM4	8572
IDH1	3417	PELP1	27043
IGF1R	3480	PHB	5245
IGFALS	3483	PIK3CA	5290
IL24	11009	PIK3R1	5295
ING3	54556	PLAGL1	5325
IRF8	3394	PLK1	5347
IRX1	79192	PRKCA	5578
JAK1	3716	PSMD10	5716
JUP	3728	PTCH1	5727
KIF14	9928	PTEN	5728
KIF7	374654	PTP4A1	7803
KLF2	10365	PTP4A3	11156
KLF4	9314	PTPRC	5788
KLF6	1316	RARRES3	5920
KRAS	3845	RASAL1	8437
LAPTM4B	55353	RASSF1	11186
LMO4	8543	RASSF8	11228
LRP1B	53353	RBM14	10432
MAL	4118	RBM3	5935
MAT2A	4144	RPA1	6117
MINA	84864	RPRM	56475
MLLT11	10962	SATB1	6304
SGMS1	259230	TP53	7157
SLC39A4	55630	TP53INP1	94241
SLC3A2	6520	TPD52	7163
SLIT2	9353	TRIM13	10206
SMARCA4	6597	TRIM35	23087
SMO	6608	TRIT1	54802
SOCS1	8651	TSC22D1	8848
SOD2	6648	TSLP	85480

SOX11	6664	TSPAN32	10077

Continued Table S4

SOX2	6657	TWIST1	7291
SOX7	83595	TWIST2	117581
SPAG9	9043	TYMS	7298
SPARCL1	8404	UCHL1	7345
SRC	6714	VEGFA	7422
STAT5A	6776	WISP1	8840
STMN1	3925	WNK2	65268
TALDO1	6888	WNT2	7472
TBX5	6910	YAP1	10413
TFG	10342	YBX1	4904
THBD	7056	ZFHX3	463

Table S5. The miRNAs corresponding to LUAD-related important genes.

**miRNA ID**	**LUAD-related important genes**
miR-101-3p	KLF6, EMP1
miR-125a-5p	SLIT2, DLC1
miR-1293	NME1
miR-142-5p	RASSF8
miR-183-5p	CAV1, SLIT2
miR-21-5p	SLIT2
miR-3065-5p	DLC1
miR-484	CAV1
miR-548l	CAV1
miR-708-5p	SLIT2
miR-93-5p	CAV1
miR-942-5p	SLIT2
miR-126-5p	DLC1

Table S6. The miRNAs corresponding to LUSC-related important genes.

**miRNA ID**	**LUSC-related important genes**
let-7a-5p	GMNN, H2AFX, FOXM1
let-7b-5p	WNK2, GMNN, H2AFX, FOXM1, CCNB1
let-7c-5p	GMNN, H2AFX
miR-100-5p	SMO, PLK1
miR-101-3p	KLF2, FHL1
miR-101-5p	STMN1
miR-103a-3p	AKAP13
miR-107	AKAP13, GPRC5A, DMBT1, CAV1

miR-1246	KLF6

Continued Table S6

miR-125a-5p	DLC1, SLIT2
miR-126-5p	DLC1
miR-127-5p	STMN1
miR-1287-5p	GMNN
miR-1295a	DUSP9
miR-134-5p	FOXM1
miR-140-5p	CCNB1, BIRC5
miR-145-5p	SOX2
miR-153-3p	ECT2
miR-15b-5p	KIF14, JUP, BIRC5
miR-181a-5p	CCNB1, BIRC5
miR-181b-5p	FAM83D
miR-181c-5p	CADM1, IRF8, ETS1
miR-183-5p	FOS, KLF6, CAV1
miR-185-5p	DUSP9
miR-224-5p	STMN1, ECT2
miR-2355-5p	NEDD9
miR-26a-5p	FAM83D, TYMS, STMN1, HSPD1, CCNB1
miR-29a-5p	CCNB1
miR-30a-5p	DCUN1D1
miR-30c-5p	DCUN1D1, CCNB1
miR-30d-5p	DCUN1D1, GMNN
miR-31-5p	FAM83D, STMN1
miR-320e	WNT2
miR-32-5p	KLF2
miR-331-3p	JUP
miR-34a-5p	WNK2, PLK1, STMN1, FOXM1, BIRC5, SOX2
miR-361-5p	AKAP13, CEACAM6
miR-425-5p	STMN1, CCNB1
miR-429	DLC1, ETS1
miR-485-5p	DLC1
miR-548l	BIRC5
miR-589-5p	FOXM1, CCNB1
miR-590-5p	OLR1
miR-642a-5p	NME1
miR-671-5p	DLC1, SLIT2
miR-7-5p	FAM83D, GMNN, TYMS, PLK1, H2AFX, CCNB1, BIRC5
miR-766-3p	DLC1
miR-766-5p	CCNB1
miR-873-5p	CAV1
miR-92a-1-5p	KLF2, GPRC5A, CAV1
miR-944	KLF2

Table S7. Regulatory pairs based on miRNA markers.

Regulation Pairs	LUAD	LUSC
miRNA-gene	80	477
miRNA-TF	13	73
miRNA-lncRNA	26	118
TF-gene	7276	41003
TF-miRNA	318	641
TF-lncRNA	14	162

Table S8. Summary of 4-node FFLs based on miRNA markers.

	I	II	III	Total
No.	4662	681	318	566
TFs	94	32	8	112
miRNAs	5	5	5	5
genes	484	276	210	486
lncRNAs	19	13	10	19
miRNA-gene	515	287	217	517
miRNA-TF	-	32	9	32
miRNA-lncRNA	24	15	11	24
TF-miRNA	153	-	9	153
TF-gene	4404	648	300	4739
TF-lncRNA	141	27	12	153
